# Temporal Stability and Practical Applicability of Velocity and Velocity-Loss Perception in Back Squat over Eight Weeks

**DOI:** 10.3390/jfmk11030345

**Published:** 2026-08-31

**Authors:** Antonio Gramazio, Emanuele Dello Stritto, Ruggero Romagnoli, Maria Francesca Piacentini

**Affiliations:** 1 Department of Human Movement and Health Sciences, University of Rome “Foro Italico”, Piazza L. De Bosis 15, 00135 Rome, Italy; a.gramazio@studenti.uniroma4.it (A.G.); emanuele.dellostritto@outlook.it (E.D.S.); 2 Department of Theoretical and Applied Sciences, eCampus University, Via Isimbardi, 10, 22060 Novedrate, Italy; rugg.romagnoli@hotmail.it

**Keywords:** velocity-based training, resistance training, autoregulation method, motor perception

## Abstract

**Background**: Subjective autoregulation methods are gaining interest among resistance-trained athletes. In particular, velocity perception (PV) and velocity loss perception (PVL) have shown promising practical applicability. This study aimed to investigate the temporal stability of PV and PVL across an eight-week period. **Methods**: Fifteen participants, after sessions of familiarization with PV and PVL, completed three test sessions: the first 48 h after the familiarization, and the others 4 and 8 weeks later. Each test session included five sets of back squats with light (55%–60%–65% 1RM) and heavy (75%–85% 1RM) loads. Light loads were executed with a 10–20–30%VL, while heavy loads with a 10–20%VL. PV accuracy was assessed using the |DeltaScore|, defined as the absolute difference between perceived velocity (Vp) and the velocity provided by the encoder (Vr): |DeltaScore| = |Vp − Vr|. PVL was measured by the |Vscore|, defined as the absolute difference between the number of repetitions perceived as correct relative to the target %VL (Np) and the repetitions that actually met it (Nr): |Vscore| = |Np − Nr|. **Results**: The results demonstrated that both PV and PVL are stable after 4 weeks at all loads and %VL (*p* > 0.05). In contrast, after 8 weeks, PV accuracy significantly decreased at all loads (*p* < 0.05; η^2^p = 0.04), whereas PVL accuracy decreased only at light loads (*p* < 0.05; η^2^p = 0.18). Moreover, PV error values remained below the previously suggested acceptability threshold of 0.09 m·s^−1^ for up to 4 weeks. This error threshold corresponds to a load variation of ~10% 1RM in the back squat. **Conclusions**: The present results support the applicability of PV and PVL in real training contexts for a maximum of 4 weeks when monitoring devices are unavailable.

## 1. Introduction

Resistance training (RT) is crucial for promoting health-related outcomes and improving performance [1,2]. These benefits rely on the proper manipulation of training variables [2]. Among these, training intensity and volume are considered the two most relevant [2]. During percentage-based training (PBT) [3,4], the most utilized method, training intensity is prescribed as a percentage of the one-repetition maximum (1RM) [5]. Meanwhile, training volume is modulated according to training intensity using a fixed number of sets and repetitions [5]. Unfortunately, PBT does not consider daily fluctuations potentially compromising the proper prescription of RT [5]. For this reason, researchers widely focused on autoregulatory approaches as alternatives [5,6,7,8,9,10,11,12,13,14,15,16,17,18,19,20,21,22]. These methods are currently divided into objective [5,22] and subjective [6,7,8,9,10,11,12,13,14,15,16,18,19,20,21] ones.

Within the objective methods, velocity-based training (VBT) represents the most widely used method for prescribing and monitoring RT, relying on the linear inverse relationship between velocity and load [5]. Specifically, the concentric velocity of the barbell during the first repetition is used to modulate intensity, while the % Velocity Loss (%VL) within the set determines the training volume [5]. Monitoring barbell velocity allows for daily load adjustment, optimizing the training prescription process [23,24]. To correctly prescribe training with VBT, specific devices are necessary, among which the most reliable are linear position transducers (LPT) [25]. LPT prices range from USD 400, for the acceptable models, to USD 2000 for those considered the gold standard [25]. For many strength and conditioning coaches, this relatively high cost makes VBT prescription impracticable, especially in team sports where more than one LPT is required simultaneously [6]. For this reason, researchers investigated some subjective autoregulative approaches such as Repetitions in Reserve [18,19,20], Velocity Perception (PV) [7,13] and Velocity Loss Perception (PVL) [11,12,13,16], as alternatives when LPT are scarce. PV is a parameter used to modulate training intensity that refers to the ability to perceive the velocity of the concentric phase of a repetition [13]. Its accuracy has been assessed using the absolute value of a DeltaScore (|DS|) calculated as the difference between perceived Velocity (Vp) and the encoder-registered Velocity (Vr) (|DS| = |Vp − Vr|) [13]. Therefore, the smaller the |DS|, the more precise the subjects. It has been demonstrated that, after a short period of combined use of LPT and a validated PV scale [9,15], Vp becomes closer to Vr both in back squats [13,14,15] and bench press [9]. Another important characteristic of PV is its stability across different conditions and over time. In fact, PV accuracy appears to be unaffected by mental [7] and physical fatigue [6] and remains stable for at least four weeks [13]. Concerning PVL, it has been employed to modulate training volume, as it consists in the ability to perceive the %VL [13]. PVL ability has been investigated in both athletes [11,12,13,16,26] and coaches [27]. Different methods to assess PVL in athletes have been studied [11,12,13,16,26]. Sindiani et al. [12] and Lazarus et al. [11] investigated the ability to perceive changes in velocity relative to the first repetition, asking participants to verbally report the perceived change. Da Silva et al. assessed the percentage of sets correctly stopped when a %VL between 15% and 30% was reached [26]. Lastly, another method used to assess PVL accuracy relies on calculating the difference between the number of repetitions participants perceived as corresponding to the target %VL, and the number of repetitions in which the %VL was actually reached [13,16]. PVL accuracy in athletes seems to be influenced by both the %VL threshold and the load, with some incongruent findings across studies [11,12,13,16,26]. Nevertheless, these discrepancies may be attributable to the different assessment methods mentioned above [11,12,13,16,26], and to the length of the familiarization. Recent studies have in fact used a longer period [13]. Moreover, PVL appears to be stable for at least four weeks [13].

Despite the substantial amount of information available on PV and PVL, it remains unclear how long the accuracy of these two parameters persists before diminishing. Therefore, the main aim of the present study is to investigate whether the accuracy of these abilities remains stable over an eight-week period. Considering the previous literature on PV and PVL trainability and stability [13], and the motor learning theories that support the stability of skill acquisition over time [28], the authors hypothesize no alterations. A secondary aim is to determine whether participants are able to detect small variations in %VL, as three closely spaced thresholds will be used (10%, 20%, and 30%VL). Specifically, this secondary aim addresses two questions: (i) whether 30%VL exhibits a similar error as for 40%VL [13] and, if so, may be considered a preferable option due to lower fatigue while maintaining a high level of PVL precision; and (ii) whether PVL error in the 10%VL condition differs from that observed in the 20%VL condition. The authors hypothesize greater accuracy at higher %VL thresholds.

## 2. Materials and Methods

### 2.1. Participants

Fifteen advanced resistance trained subjects [29] (10 males, 5 females, age: 23.20 ± 2.30 years, height: 1.73 ± 0.07 m, body mass: 72.9 ± 11.3 kg, 4.6 ± 2.3 years of experience; 1RM/BW 1.6 ± 0.3), with no previous VBT experience, participated in the present study. The study’s inclusion criteria were at least 2 years of experience in RT, particularly with back squats, and an age between 18 and 35 years. Subjects were excluded if they did not meet these criteria, had previous experience with VBT, or had a current or prior injury within the previous two years. Before starting the first session of the study, participants read and signed the informed consent and data processing forms. The study protocol was developed in accordance with the current revision of the Helsinki Declaration, which aims to ensure the protection of the rights, integrity, and well-being of the subjects involved in experiments, and was approved by the Institutional Review Board (CAR 161/2023). Participants could withdraw from the study at any time without providing justification and without any consequences.

### 2.2. Design

Participants completed six training session separated by at least 48 h. Each training sessions started with a standardized warm-up consisting of 5 min of self-paced cycling, followed by each subject’s personal mobility routine. During the first three sessions, participants were familiarized with VBT, focusing on performing the concentric phase of each repetition as fast as possible and on the sensations perceived during the repetitions. The last three sessions were the test sessions. The fourth was performed within 72 h from the last familiarization session (T1), while the fifth and the sixth sessions were conducted four (T2) and eight (T3) weeks after the fourth session, respectively. During each test session, participants performed five sets of back squats in a randomized and counterbalanced order. Each session included three sets with light loads (~60% 1RM), executed at 10%, 20%, and 30%VL, and two sets with heavy loads (~80% 1RM), executed at 10% and 20%VL. The decision to avoid 30%VL in the heavy condition was based on previous results showing similar PVL errors between 20% and 40%VL at 80% 1RM [13], and aimed to prevent excessive fatigue. Light loads corresponded to ~55%, ~60%, and ~65% 1RM, while heavy loads corresponded to ~75% and ~85% 1RM. Each %1RM was performed using one specific %VL, which was randomly assigned during the first session and consistently maintained across all testing sessions. However, as stated above, the order of execution was randomized and counterbalanced within each testing session. During the test sessions, the loads were concealed and participants were not aware of the amount to be lifted. Participants were instructed to refer the perceived velocity of the first repetition and to stop the set when they believed they had reached the target %VL indicated prior to starting the set. Before starting the test sessions, participants were reminded to perform every repetition as fast as possible during the concentric phase. Nevertheless, no verbal encouragement was provided, in order to avoid influencing their perception.

### 2.3. Familiarization Sessions

The first three sessions were designed to familiarize participants to move the barbell as fast as possible. Moreover, they were familiarized with repetition velocity, %VL, PV and PVL. The familiarization protocol was designed in accordance with previous studies showing that a familiarization period of three sessions resulted in comparable PV accuracy to that observed following longer exposure to VBT training feedback [10,13]. During the sessions, the mean propulsive velocity (MPV) of each repetition was recorded and displayed via audio and visual feedback using “Vitruve” (SPEED4LIFTS S.L., Madrid, Spain) and its software 2.4.3. Moreover, after each set, participants visualized each repetition on the validated PV squat scale [15]. In the first session, before starting the warm-up, participants signed the informed consent, and their essential data were collected, including height, body mass, age, and RT experience. Familiarization sessions consisted of seven or eight sets of squats. The first two (or three) sets were designed to complete the warm-up process, with the load progressively increasing. The remaining sets in each session were used to familiarize participants with different velocities. During session one, sets with MPV between 0.6 and 0.4 m/s were performed. In session two, MPV ranged from 0.8 to 0.6 m/s. The third session included three sets with MPV ~0.7 m/s and two sets with MPV ~0.5 m/s. Each load was performed at 10%, 20%, and 30%VL if the MPV of the first repetition was above 0.5 m/s, or only at 10% and 20%VL if below 0.5 m/s. During familiarization, participants received auditory and visual feedback on the MPV of each repetition and the %VL (indicated by a different sound and color on the Vitruve app). After each set, participants observed the velocity attained on the PV scale and were instructed to focus on the effort and feeling associated with each %VL, rather than on the number of repetitions. During this phase, data on MPV and the corresponding load were collected. At the end of these three sessions, these data were used to establish the individual load–velocity profile and to estimate the 1RM, which allowed for the determination of the loads for the subsequent test phase.

### 2.4. Test Sessions; PV and PVL Assessment

The three test sessions were performed four weeks apart with the last one conducted 8 weeks after the first one. Between test sessions, participants continued performing their normal training routine, consisting of at least three training sessions per week, one of which included the free-weight back squat. Compliance with their regular training routine between test sessions was verbally self-reported by the subjects. Moreover, all participants avoided performing VBT sessions or receiving further velocity feedback during this period. During these sessions the accuracy of PV and PVL for each subject was assessed. Between the different sessions, participants continued regular RT using PBT, without focussing on PV and PVL, and without receiving feedback from LPT or using the PV scale.

PV characteristics were calculated using the delta score (DS) and its absolute value (|DS|), as done previously [13]. The DS, calculated as the difference between Vp and Vr (DS = Vp − Vr) was used to determine the direction of the error, with positive values indicating an overestimation of MPV and negative values indicating underestimation. The |DS|, that describes the absolute differences between Vp and Vr (|DS| = |Vp − Vr|), was used to quantify the magnitude of the error, avoiding any compensation caused by the presence of over and underestimations.

PVL accuracy was assessed as previously described [13], using a Vscore and |Vscore|. The Vscore was calculated as the difference between the number of repetitions performed by the participant (Np) and the number of repetitions required to effectively reach the prescribed VL threshold (Nr) (Vscore = Np − Nr). Accordingly, the |Vscore| corresponded to the absolute value (|Vscore| = |Np − Nr|). As described for DS and |DS|, Vscore and |Vscore| were used to analyze the direction (overestimation or underestimation) and the magnitude of error, respectively.

**Figure 1:**
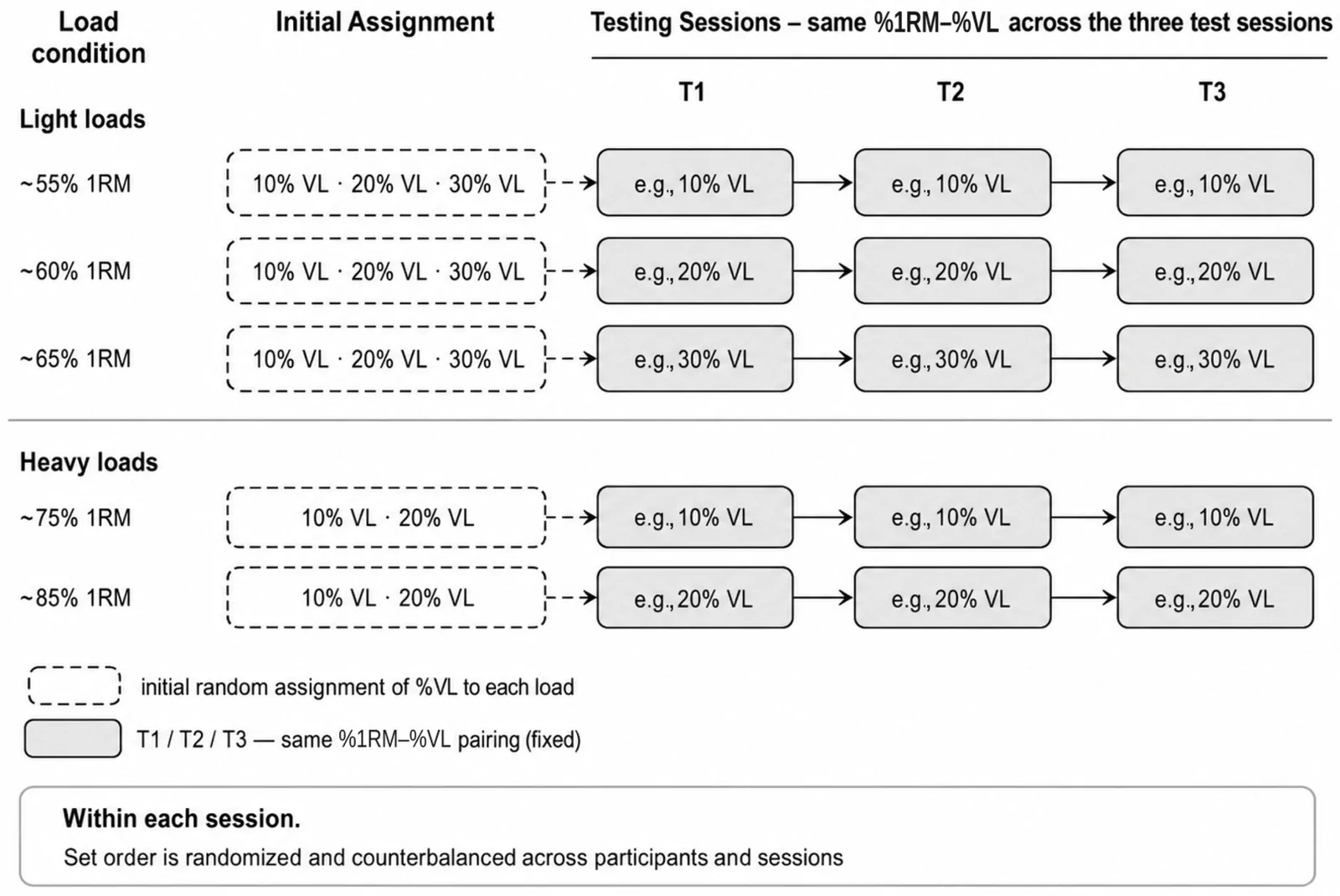
Representation of the random assignment, for each participant, of a %VL threshold to each %1RM load prior to T1, and the maintenance of this individualized %1RM–%VL combination across the three test sessions. T1: Test session 48–72 h after the familiarization protocol; T2: Test session 4 weeks after T1; T3: Test session 8 weeks after T1. Light Loads: loads between ~55% 1RM and ~65% 1RM; Heavy Loads: loads between ~75% 1RM and 85% 1RM; VL: Velocity loss.

During the test sessions, participants performed five sets of back squats. Three of these sets were performed with light loads corresponding to ~55%, ~60%, and ~65% 1RM, while the other two sets were performed with heavy loads of ~75% and ~85% 1RM. The light-load sets were executed at %VL of 10, 20, and 30%, whereas the heavy-load sets were performed at 10% and 20%VL. Before the first test session, each load was randomly assigned to a specific %VL. This procedure was performed individually for each participant. Once assigned, the same %1RM–%VL combinations were maintained across all three test sessions for each load. The order of execution was randomized and counterbalanced in every different test session. The decision to slightly vary the loads was made to avoid learning effects associated with specific loads across sets and across the three testing sessions. A clear representation of test and re-test procedures is shown in Figure 1.

During the sessions, the loads were concealed to be unidentifiable by participants. Prior to the five prescribed sets, participants were asked to perform at least three warm-up sets. They were then informed that, during the test, they would need to be ready to lift any load, including their 1RM. Moreover, they were instructed to always approach the barbell as though preparing to lift their 1RM.

Lastly, participants were instructed to verbally report the Vp of the first repetition and to end the set when they believed they had reached the required VL threshold (Np). In cases where repetitions were underestimated and the set was stopped before reaching the %VL, the VL slope during the set was used to estimate the number of repetitions required to reach the VL threshold (Nr).

### 2.5. Statistical Analysis

The distribution of the data was assessed using the Shapiro–Wilk test for DS, |DS|, Vscore, and |Vscore|, whereas the Kolmogorov–Smirnov test was applied to Vp, Vr, Np, and Nr.

For the analysis, loads were grouped as light loads (55%, 60%, 65% 1RM) and heavy loads (75%, 85% 1RM). This grouping was adopted to increase the number of observations available for analysis within each load condition, and because the loads within each load condition are associated with similar movement velocities.

Due to the non-normal distribution of the data, two separate Aligned Rank Transform (ART) ANOVAs were conducted, one for DS and one for |DS|. In both analyses, time (T1, T2, and T3) and load (light and heavy) were included as within-subject factors. The main effects of time and load, as well as their interaction (time × load), were evaluated. When statistically significant effects were observed, appropriate post hoc analyses were performed.

Regarding the |Vscore| considering the non-normal distribution of the data and the unbalanced experimental design (30%VL condition present only in the light load), two different ART ANOVA were performed. The first one was used to analyze the effects of time (T1, T2, and T3), two %VL (10%, and 20%), and load (light, and heavy). Moreover, the effects of doubles (time × load; time × %VL; %VL × Load) and triple interaction (time × load × %VL) were assessed. The second one was performed only on light loads and were needed to evaluate the differences between the three %VL conditions (10%, 20%, and 30%) across time (T1, T2, and T3). Furthermore, the effects of the interaction were assessed (time × %VL). For the Vscore used to analyze the direction of error, the same exact procedure employed for |Vscore| was utilized. In all those analyses, whether a statistic significance emerged, the subsequent post hoc analysis was performed.

For both main and post hoc analysis, significance was set at alpha = 0.05. To control for multiple comparisons, *p*-values were Bonferroni-adjusted by multiplying the observed *p*-value by the number of comparisons (p_adj_). Only p_adj_ below 0.05 were considered significant.

Effect sizes were interpreted as follows [30]: for partial eta-squared (η^2^p), values of 0.01, 0.06, and 0.14 were considered small, medium, and large, respectively; for post hoc pairwise comparisons, effect sizes (r) were interpreted with values of 0.10, 0.30, and 0.50 considered small, medium, and large, respectively.

The descriptive values of non-normally distributed data will be presented as Median and Interquartile Ranges (IQR) (Table 1 and Table 2). Moreover, to facilitate a clearer interpretation of the results, means and standard deviations (SDs) (Table 1 and Table 2) will also be reported when necessary.

Vp and Vr were normally distributed. Therefore, their agreement was assessed using Bland–Altman plots and the Intra-Class Correlation (ICC; 2.1), respectively, for the three test sessions and for each load. Additionally, simple linear regression between Vp and Vr was performed, both grouping the data by load and by session, to determine the coefficient of determination (R^2^). The magnitudes of ICC were classified as follows: poor (<0.5), moderate (0.5–0.75), good (0.75–0.9), and excellent (>0.9). Instead, Since Np and Nr were not normally distributed, their agreement and correlation were assessed using Bland–Altman plots, Kendall’s tau (τ), and ICC, both for each test session and grouped by load. Moreover, Theil–Sen regression was performed to determine Sen’s slope (β_1_) for individual sessions and for data grouped by load.

The data were analyzed using “R Core Team (2025). _R 4.6.1: A Language and Environment for Statistical Computing_. R Foundation for Statistical Computing, Vienna, Austria. <https://www.R-project.org/>, (accessed on 15 may 2026).” The following R packages were employed: “ARtool” for conducting ART ANOVA, “emmeans” for calculating estimated marginal means and post hoc comparisons, “effectsize” for computing standardized effect magnitudes, “dplyr” and “ggplot2” for data manipulation and graphical representation, respectively, and “Rcmdr” for dataset management.

An a priori power analysis was conducted using G*Power 3.1.9.7. The “ANOVA: Repeated Measures, within factors” F test was selected. Based on previous results, the analysis was based on an effect size of 0.31 [13], an α level of 0.05, and a statistical power of 0.80 with six measurements as within-subject factors (three time × two loads). To adopt a conservative approach, a correlation of 0.50 among repeated measures and a non-sphericity correction of 0.80 were assumed. The resulting estimated sample size was 15 participants in total.

## 3. Results

### 3.1. Delta Score and Absolute Delta Score

The ART ANOVA for |DS| revealed significant main effects for time (F(2) = 4.64, *p* = 0.01, η^2^_p_ = 0.04), but not for load and time × load interaction. Post hoc analysis revealed significant differences only between T1 and T3 (p_adj_ < 0.01; r = 0.207). The DS showed no effects for time, load, and their interaction.

**Figure 2:**
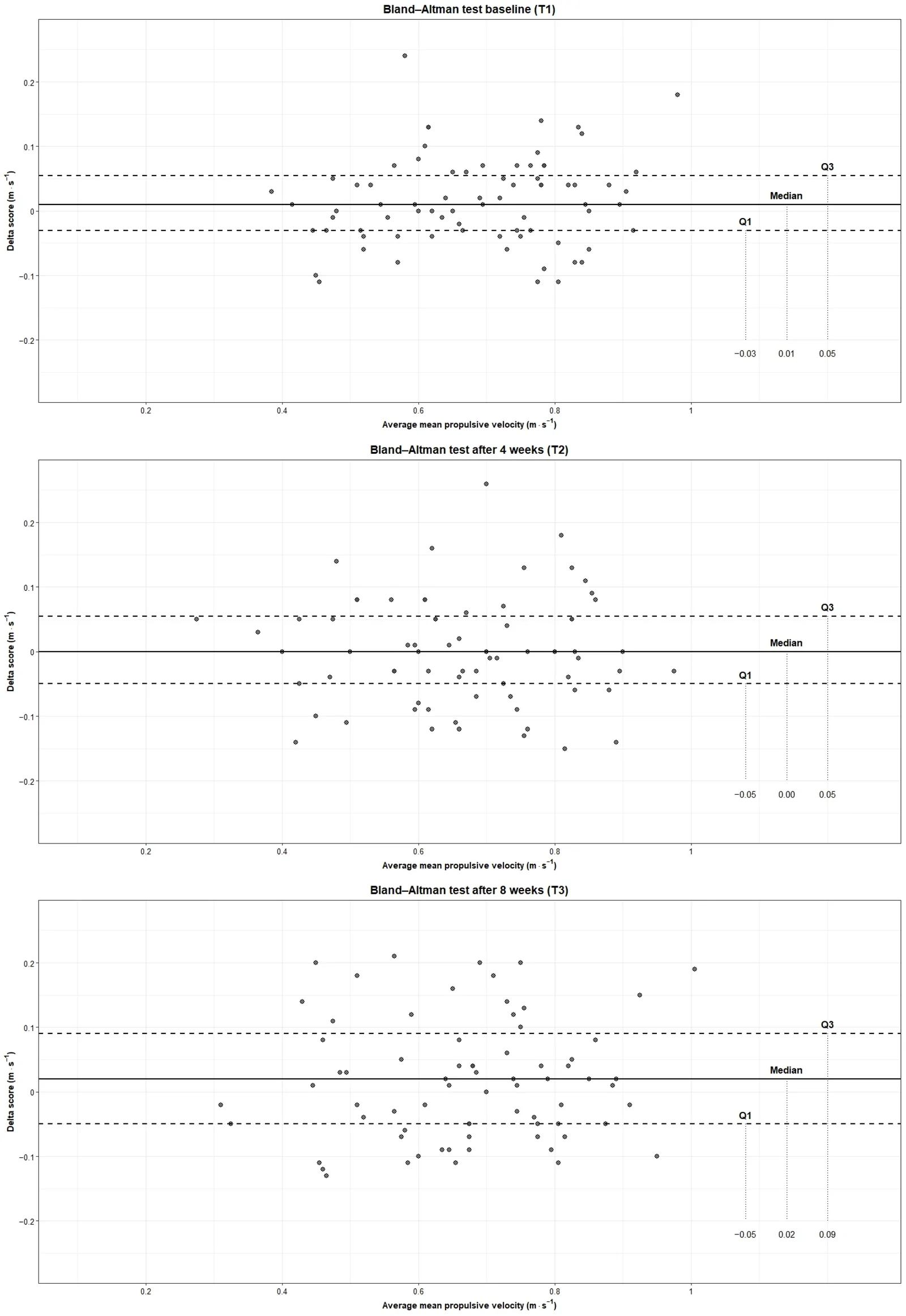
Delta score: differences between perceived velocity and real velocity (Vp − Vr); Vr: real velocity; Vp: perceived velocity. T1: Test session 48–72 h after the familiarization protocol; T2: Test session 4 weeks after T1; T3: Test session 8 weeks after T1.

ICC showed good within-session reliability of measurement at T1, T2, and T3. Moreover, a high correlation was demonstrated by the R^2^ values at all time points. Instead, moderate and poor-to-moderate reliability was shown by ICC for heavy and light load conditions, respectively. The R^2^ values resulted in moderate and weak values for heavy and light load conditions (Table 3), respectively. Moreover, the results of the Bland–Altman plot are shown in Figure 2.

### 3.2. Vscore and Absolute Vscore

The ART ANOVA for |Vscore|, including load, time, and VL as main factors showed significant effects for load (F(1) = 34.01, *p* < 0.01, η^2^p = 0.18) and VL (F(1) = 4.24, *p* = 0.04, η^2^p = 0.06), but only a trend for time (F(2) = 2.52, *p* = 0.08, η^2^p = 0.03). Moreover, significant interactions were found for time × load (F(2) = 5.29, *p* > 0.01, η^2^p = 0.06), and load × VL (F(1) = 4.18, *p* = 0.04, η^2^p = 0.03). Post hoc analysis revealed significant differences between heavy and light conditions at T3 (p_adj_ < 0.01, r = 0.342), and for both the %VL threshold of 10% and 20%VL. Regarding the direction of the error, Vscore analysis revealed significant main effects for time (F(2) = 9.97, *p* = 0.02, η^2^p = 0.05), load(F(1) = 13.70, *p* < 0.01, η^2^p = 0.08), VL (F(1) = 4.08, *p* < 0.05, η^2^p = 0.03), and time × load interaction (F(2) = 3.43, *p* = 0.04, η^2^p = 0.04). Post hoc analysis revealed significant differences only between T1 and T3 (p_adj_ = 0.02, r = 0.218), whereas trends were observed between the heavy and light conditions at T3 (p_adj_ = 0.06, r = 0.227) and between the heavy condition at T1 and light condition at T3 (p_adj_ = 0.07, r = 0.225).

Considering only the light condition, the ART ANOVA performed for |Vscore|, including the 30%VL threshold, showed a significant main effect only for time (F(2) = 5.90, *p* < 0.01, η^2^p = 0.10). In addition, a trend toward significance was observed for VL (*p* = 0.06, η^2^p = 0.05). Post hoc analysis revealed a significant effect only between T2 and T3 (p_adj_ < 0.01, r = 0.301). The direction of the error’s analysis revealed significant main effect only for VL (F(2) = 11.32, *p* < 0.01, η^2^p = 0.17). Post hoc comparisons showed significant differences between the 10%VL and the 20%VL thresholds (p_adj_ < 0.01, r = 0.352) and between 20%VL and 30%VL thresholds (p_adj_ < 0.01, r = 0.372).

The reliability analysis showed excellent values between Np and Nr at T1 and T2, whereas only good within-session reliability was observed at T3 (ICC = 0.864). Moreover, Kendall’s tau revealed strong correlations among measurements at all time points. In addition, a perfect β = 1 emerged from all Theil–Sen regression analysis. Moreover, both loads showed good reliability (light loads ICC: 0.878; heavy loads ICC: 0.865), but strong correlation between measurements and a perfect slope (β = 1) emerged from Kendall’s tau and Theil–Sen regression analysis, respectively (Table 4).

## 4. Discussion

Perception skills, namely PV and PVL, offer a practical alternative for autoregulating VBT sessions when monitoring devices are scarce. However, evidence on how long these parameters remain stable is limited to a four-week period [13]. The present study addressed this gap by examining PV and PVL stability across an eight-week period at different loads and %VL thresholds. Moreover, another considerable aim was the investigation of PVL accuracy at VL threshold of 10%VL and 30%VL.

### 4.1. Delta Score and Velocity Perception

This study is the first to investigate the stability of PV across an eight-week period. We found PV stability after four weeks as previously reported [13]. In contrast, after eight weeks (T3), a significant increase in |DS| was found compared to T1. However, delving deeper into the analysis, although the difference between T1 and T2 did not reach statistical significance (*p* = 0.24), the data showed a decreasing pattern in PV accuracy across all time points, with medians of 0.04 m·s^−1^ (IQR: 0.05), of 0.07 m·s^−1^ (IQR: 0.08), and of 0.09 m·s^−1^ (IQR: 0.09) at T1, T2, and T3, respectively. Notably, even between T1 and T2 a 75% reduction in accuracy and greater error dispersion, as indicated by the IQR (0.05 m·s^−1^ vs. 0.08 m·s^−1^) and Bland–Altman plot (Figure 2), were observed. Thus, even if this change was not statistically significant, it may still have practical relevance, potentially compromising load selection and consequently shifting the training focus.

Regarding PV characteristics over different loads, no differences between light and heavy loads (Heavy median: 0.05 m·s^−1^ IQR 0.07 vs. Light median: 0.05 m·s^−1^ IQR 0.07) emerged. These results differ from previous findings showing higher accuracy at heavy loads [6,10,13]. This difference may be attributed to the absence of a 1RM test in the present study, which may have limited participants’ familiarity with very slow velocities (<0.4 m·s^−1^), accounting for the observed discrepancies with previous investigations [6,10,13].

Lastly, it has to be noted that this is the first study in which participants showed an error below the previously suggested acceptability threshold of 0.09 m·s^−1^ [13], as errors above this value correspond to an approximate variation of ~10% 1RM [31], which could lead to a meaningful alteration of the training focus. This outcome, and the absence of differences between heavy and light loads, may suggest that when working with athletes with high PV accuracy (|DS| values below 0.09 m·s^−1^), as in the present study, the use of PV for VBT prescription at all loads could be feasible whenever LPTs are absent or scarce. Considering the present results, we suggest that a prior assessment of PV accuracy may help determine its suitability for prescribing training intensity. Moreover, it would be worth understanding the mechanisms behind these individual differences in PV accuracy. In the authors’ opinion, they could, at least in part, reflect differences in underlying sensory mechanisms [32,33,34]. Indeed, it should be highlighted that the detection of changes in velocity appears to depend on both proprioceptive and exteroceptive afferent input [32,33,34]. However, no study has yet examined whether individual sensory acuity is directly correlated with PV accuracy. Future studies should address this gap, as a better understanding of this relationship could help identify which athletes can more reliably use PV for VBT training prescription.

### 4.2. Vscore and Velocity Loss Perception

The results of our study confirm a stability of PVL across 4 weeks [13] over all %VL and loads. In contrast, a significant increased error at T3 emerged for light loads only. These findings suggest providing athletes with an additional familiarization period between four and eight weeks after the first one at least for light loads. In fact, an error of two or three repetitions may result in a shift in training focus and compromise the intended training goal.

In the present study, load strongly influenced the PVL accuracy (η^2^p = 0.18), with subjects being more precise in heavy load conditions at each time point, particularly at T3 (p_adj_ < 0.01). Regarding this aspect, there is no real consensus in the literature. Indeed, some studies showed better PVL accuracy with heavy loads (80% 1RM) [13,26], while others reported better accuracy with light loads (50–60% 1RM) [16]. Discrepancies in familiarization strategies could have determined these differences. Specifically, the study reporting better accuracy at lower loads (50–60% 1RM) performed only one familiarization session [16], whereas the other two studies provided %VL feedback across multiple training sessions [13,26]. The authors hypothesize that the extended familiarization could have enhanced PVL accuracy at heavy loads by increasing participants’ awareness of the relationship between the point of failure and %VL when heavy loads are used. In fact, during sets with heavy loads, participants often came close to muscular failure, a condition already suggested to be favorable for %VL threshold recognizability [13].

The analyses conducted showed that PVL is slightly influenced by %VL, with a small- to medium-sized effect (η^2^p = 0.03) when both loads were considered, with lower error at 10%VL than at 20%VL. These findings slightly differ from most recent ones [13], which indicated greater PVL accuracy at higher VL thresholds (40%VL compared to 20%VL) with a load of 60% 1RM, suggesting that the closer participants are to muscular failure, the easier it becomes to detect %VL. The authors propose two possible explanations for the present findings and their discrepancy with previous results [13]. First, the load%VL combinations adopted in the present study likely resulted in sets being performed relatively far from muscular failure [35], as supported by the average MPV of the last repetition (light load, 30%VL ~0.49 m·s^−1^; heavy load, 20%VL ~0.41 m·s^−1^). Second, the greater PVL accuracy observed at 10%VL compared with 20%VL, may be explained by a learning effect related to the number of repetitions performed. Indeed, authors speculate although participants were strongly instructed to focus on sensations related to decreases in velocity and fatigue at different %VL thresholds, it could be hypothesized that during familiarization they learned that 10%VL was typically reached after only a few repetitions (~five repetitions). This prior experience may have inadvertently masked errors, as participants may have stopped the sets not solely based on their perception but also by relying on the number of repetitions performed. Therefore, they may have avoided performing more repetitions than usual, thereby reducing the likelihood of large errors. This interpretation, however, remains speculative, as the present study did not directly assess participants’ repetition-counting strategies during the sets. Moreover, it has to be noted that the aforementioned results may have been influenced by some outliers that emerged in the light-load condition (maximum error at T1: 10%VL = 2, 20%VL = 6; at T3: 10%VL = 3, 20%VL = 7), as shown by the scatter plot (Figure 2).

Lastly, in light of the practical relevance that this article aims to provide, while acknowledging the limitations of formulating practical suggestions based solely on descriptive data, a closer analysis of the descriptive data may help in formulating more useful recommendations for practical application (Table 2). Considering the mean error between zero and one repetition, the use of PVL might be suggested for loads above 75% 1RM for all %VL at least for eight weeks. In contrast, for loads below 70% 1RM between four and eight weeks an increasing error trend was observed potentially suggesting the need for new familiarization. Moreover, for light loads, only the 10%VL seems reliable. However, it should be emphasized that an error of two or three repetitions, as may occasionally occur at this VL threshold, could have a substantial impact on the total training volume, considering that the average number of repetitions required to reach the 10%VL threshold is approximately five. Therefore, although PVL may represent a valid alternative to the use of LPT, caution is required when applying PVL at 10%VL with load ~60% 1RM. With respect to the 20% and 30%VL thresholds, in the absence of LPT, the fixed method (i.e., the traditional method) should be preferred over PVL-based prescriptions, given the magnitude of the error, its variability, and the presence of outliers, even shortly after familiarization, which could compromise the focus of training.

**Figure 3:**
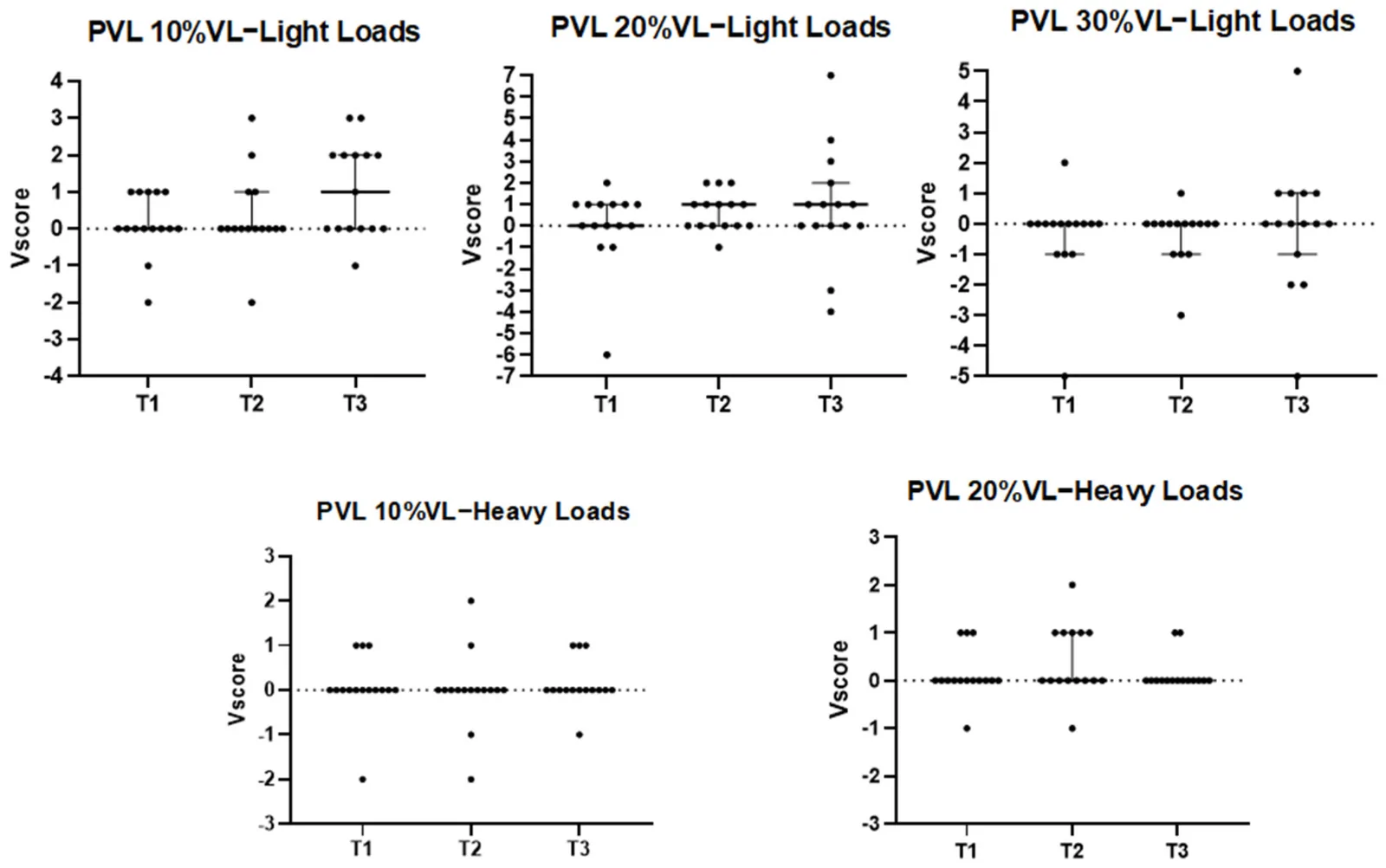
Vscore: differences between Nr and Np (Nr − Np); Np: number of repetitions completed at the perceived threshold; Nr: number of repetition where the subject actually reached the required threshold; T1: Test session 48–72 h after the familiarization protocol; T2: Test session 4 weeks after T1; T3: Test session 8 weeks after T1; Light Loads: loads between ~55% 1RM and ~65% 1RM; Heavy Loads: loads between ~75% 1RM and 85% 1RM.

Notably, interesting findings emerged from the analysis of the direction of the error. Indeed, a prevalence of overestimation of the number of repetitions required to reach the VL threshold was observed for %VL below 30% at all time points, as indicated by the Vscore (Figure 3). These findings should be taken into account whenever PVL is used as a parameter for prescribing training volume, as a consistent overestimation of the number of repetitions to be performed could lead to excessive fatigue, potentially compromising training adaptations.

## 5. Limitations

It should be highlighted that the unbalanced number of observations between light and heavy load conditions may have partially masked the results for the heavy load and their comparison with the light load, potentially reducing the statistical power of the analysis and the reproducibility of the findings. Indeed, as mentioned in the discussion regarding the PV, the present investigation found an unusually equal precision between loads at the first test session. However, the authors hypothesize that including a 1RM testing session could have reduced the measurement error for heavy loads, thereby confirming differences previously reported. However, the high accuracy at all loads found in this study could also be responsible for the lack of differences, as a ceiling effect may have occurred. Moreover, the relatively small sample size, differences in RT experience (4.6 ± 2.3 years) and unexplored sex-related differences in PV and PVL accuracy could have influenced the present results.

## 6. Conclusions

This study is the second to investigate the temporal stability of PV and PVL, extending the observation period from four to eight weeks. While confirming the stability of both parameters after four weeks, a general tendency for decreased accuracy in PV and PVL was observed after eight weeks. Therefore, authors hypothesize that a new familiarization period could be needed between four and eight weeks after the first one to maintain similar perceptual levels for both parameters.

For the first time, PV accuracy reached levels well below the acceptability threshold (|DS|~0.09 m·s^−1^) [13], with errors of 0.04 m·s^−1^ at baseline and 0.07 m·s^−1^ after four weeks. These results have considerable practical implications, suggesting PV as a valid method for prescribing intensity in populations with high perceptual abilities.

Regarding PVL, it was shown to be a valid parameter for prescribing training volume for at least four weeks when using loads above 75% 1RM. For loads below 70% 1RM, caution should be taken when using PVL at thresholds of 10%VL, 20%VL, and 30%VL.

However, these findings should be interpreted with caution, as they are based on a relatively small sample of resistance-trained individuals with heterogeneous RT experience (4.6 ± 2.3 years) and including both sexes; therefore, their generalizability to novice lifters, elite athletes, or different exercises remains to be established. Given these limitations and considering that a direct comparison between objective and subjective autoregulation methods goes beyond the aims of this study, LPT should still be preferred over PV and PVL whenever available.

## Figures and Tables

**Table 1:** Delta score: differences between perceived velocity and real velocity (Vp − Vr); Vr: real velocity; Vp: perceived velocity. T1: Test session 48–72 h after the familiarization protocol; T2: Test session 4 weeks after T1; T3: Test session 8 weeks after T1. Light Loads: loads between ~55% 1RM and ~65% 1RM; Heavy Loads: loads between ~75% 1RM and 85% 1RM.

Absolute Delta Score (m·s^−1^)						
Loads	Light			Heavy		
Time	T1	T2	T3	T1	T2	T3
Median (IQR)	0.05 (0.04)	0.05 (0.09)	0.06 (0.08)	0.04 (0.07)	0.06 (0.07)	0.08 (0.08)
Mean ± sd	0.06 ± 0.04	0.08 ± 0.09	0.09 ±0.09	0.05 ±0.05	0.07 ± 0.05	0.08 ± 0.06

**Table 2:** Absolute Vscore: absolute differences between Nr and Np (Nr − Np); Np: number of repetitions completed at the perceived threshold; Nr: number of repetition where the subject actually reached the required threshold; T1: Test session 48–72 h after the familiarization protocol; T2: Test session 4 weeks after T1; T3: Test session 8 weeks after T1; Light Loads: loads between ~55% 1RM and ~65% 1RM; Heavy Loads: loads between ~75% 1RM and 85% 1RM.

**(A). Absolute Vscore (Number of Repetitions) Light Loads**												
**Time**	**T1**				**T2**				**T3**			
%VL	10%	20%		30%	10%	20%		30%	10%	20%		30%
Median (IQR)	0.0 (1.0)	1.0 (1.0)		0.0 (1.0)	0.0 (1.0)	1.0 (1.0)		0.0 (1.0)	1.0 (2.0)	1.0 (3.0)		1.0 (1.5)
Mean ± sd	0.5 ± 0.6	1.1 ± 1.5		0.7 ± 1.3	0.6 ± 1.0	0.8 ± 0.8		0.5 ± 0.8	1.2 ± 1.1	1.8 ± 2.0		1.2 ± 1.7
**(B). Absolute Vscore (Number of Repetitions) Heavy Loads**												
**Time**	**T1**				**T2**				**T3**			
%VL	10%		20%		10%		20%		10%		20%	
Median (IQR)	0.0 (0.5)		0.0 (0.5)		0.0 (0.5)		0.0 (1.0)		0.0 (0.5)		0.0 (0.0)	
Mean ± sd	0.3 ± 0.6		0.3 ± 0.5		0.4 ± 0.7		0.5 ± 0.6		0.3 ± 0.5		0.1 ± 0.4	

**Table 3:** Intra-class correlation coefficient (ICC) and coefficient of determination (R^2^) between Vp and Vr across time (**A**) and load (**B**). CI: 95% Confidence Interval. Vr: real velocity; Vp: perceived velocity. T1: Test session 48–72 h after the familiarization protocol; T2: Test session 4 weeks after T1; T3: Test session 8 weeks after T1; Light Loads: loads between ~55% 1RM and ~65% 1RM; Heavy Loads: loads between ~75% 1RM and 85% 1RM.

**(A)**						
	**T1**		**T2**		**T3**	
	**ICC**	**R^2^**	**ICC**	**R^2^**	**ICC**	**R^2^**
Vp, Vr	0.885 (CI: 0.823–0.926)	0.793	0.794 (CI: 0.693–0.864)	0.638	0.763 (CI: 0.610–0.841)	0.612
**(B)**						
	**Light Loads**			**Heavy Loads**		
	**ICC**		**R^2^**	**ICC**		**R^2^**
Vp, Vr	0.518 (CI: 0.380–0.683)		0.295	0.728 (CI: 0.615–0.812)		0.532

**Table 4:** Intra-class correlation coefficient (ICC), Kendall’s tau (τ), and Sen’s slope (β_1_) between Np and Nr across time (**A**) and loads (**B**); CI: 95% Confidence Interval. Np: number of repetitions completed at the perceived threshold; Nr: number of repetition where the subject actually reached the required threshold; T1: Test session 48–72 h after the familiarization protocol; T2: Test session 4 weeks after T1; T3: Test session 8 weeks after T1; Light Loads: loads between ~55% 1RM and ~65% 1RM; Heavy Loads: loads between ~75% 1RM and 85% 1RM.

**(A)**									
	**T1**			**T2**			**T3**		
	**ICC**	**τ**	**β**	**ICC**	**τ**	**β**	**ICC**	**τ**	**β**
Np, Nr	0.937 (CI: 0.902–0.960)	0.890	1	0.960 (CI: 0.938–0.975)	0.844	1	0.865	0.865 (CI: 0.754–0.905)	1
**(B)**									
	**Light Load**				**Heavy Load**				
	**ICC**		**τ**	**β**	**ICC**		**τ**		**β**
Np, Nr	0.878 (CI: 0.833–0.912)		0.796	1	0.866 (CI: 0.801–0.911)		0.780		1

## Data Availability

Data are available on request due to restrictions (privacy).

## Abbreviations

The following abbreviations are used in this manuscript:

RT	Resistance Training
PBT	Percentage-Based Training
1RM	One-Repetition Maximum
VBT	Velocity-Based Training
%VL	Percentage of Velocity Loss
LPT	Linear Position Transducers
PV	Velocity Perception
PVL	Velocity Loss Perception
DS	Delta Score
Vp	Velocity Perceived
Vr	Real Velocity
MPV	Mean Propulsive Velocity
Np	Number of repetitions Performed by participant
Nr	Number of repetitions required to effectively reach the %VL
ART	Aligned Rank Transform
T1	Baseline Test
T2	Test after 4 weeks
T3	Test after 8 weeks
ICC	Intra-Class Correlation
R^2^	Coefficient of Determination
τ	Kendall’s tau
β_1_	Sen’s Slope
IQR	Interquartile Range
p_adj_	p adjusted for Bonferroni correction
CI	95% Confidence Interval
